# Customizing the electric field of metalens with high degrees of freedom based on neural network

**DOI:** 10.1515/nanoph-2025-0032

**Published:** 2025-04-14

**Authors:** Quansheng Zhang, Di Guo, Changsheng Shen, Zhaofu Chen, Hehong Fan, Changgui Lv, Qilong Wang, Ningfeng Bai

**Affiliations:** School of Electronic Science and Engineering, 12579 Southeast University, Nanjing 210096, China

**Keywords:** neural network, metalens, custom electric field, high degree of freedom

## Abstract

The metasurfaces are able to flexibly control electromagnetic waves. However, their design necessitates strict phase matching, which requires high computational load, and its control method has low flexibility. This paper proposes a novel method for the customization of the electric field of metalens with high degrees of freedom based on an improved Pixel Generative Adversarial Network (I-pixGAN). This I-pixGAN can design the phase distribution of the metalens according to the customized target electric field, and the constructed metalens based on this phase can modulate the target electric field. The results show that the proposed I-pixGAN can arbitrarily control the position of the focus in three-dimensional space, where the focus position is offset within one wavelength. Therefore, it is not need to strictly follow the electric field distribution characteristics, and it can flexibly construct unknown electric field, realizing a highly free electric field customization method in three-dimensional space, providing a new way for electric field operation. This paper promotes the development of electric focusing systems, not only improving focusing accuracy but also enhancing system flexibility and promoting the automation and intelligence of optical and terahertz systems.

## 1 Introduction

A metasurface can accurately modulate electromagnetic waves, and its process is straightforward. It is composed of several unit structures, which regulate the phase, amplitude, and polarization state of electromagnetic waves by introducing phase mutations [1], [2]. The metasurfaces have a wide application range, including the metalens [3], [4], [5], [6], [7], [8], [9], holographic metasurfaces [10], [11], [12], [13], [14], [15], [16], vortex beam metasurface [17], [18], [19], [20], [21], [22], and absorbers [23], [24], [25], [26], [27]. Although they have very high electromagnetic wave control ability, their mainstream design is based on the generalized Snell’s law, the unit structure successively satisfies the phase response, and its implementation lacks flexibility [28], [29]. In the electromagnetic wave regulation field, the generalized Snell’s law provides reliable theoretical basis and calculation means. However, they limit the innovation and diversity of the design. To solve these problems, Deng et al. proposed inverse designed Jones matrix metasurface polarizers [30], [31]. Due to the improvement of the computing power and the development of the machine learning technology, the neural networks became able to control the electric fields [32], [33], [34], [35], [36].

In recent years, the combination of neural networks and metasurfaces has been widely studied. For instance, Jiang et al. [37] performed an inverse design of metasurfaces and used a neural network to accurately predict the unit structure composed of six geometric parameters, in order to meet their phase requirements. An et al. [38] proposed a Generative Adversarial Network (GAN) that can generate unit structures according to the requirements. To generate the unit structure, they input its electromagnetic response into the GAN. The experimental results showed that this network meets the unit structure requirements of various metasurfaces. Wang et al. [39] applied the GoogLeNet-Inception-V3 network model to the metasurface design and used the image recognition network for the prediction of the metasurface phase. This allows to accurately predict the phase of the unit structures and to establish a unit structure library for the design of metasurfaces. Li et al. [40] proposed an inverse method of superoscillation focusing metalens based on the complex amplitude encoding technology, which converts the complex transfer function of a given super-oscillation focusing field into a pure phase transfer function. The implementation of this method is straightforward, and it has a high focused efficiency. Jiang et al. [41] proposed an efficient end-to-end inverse design framework for multi-wavelength achromatic metalens. Their experiments demonstrated that this inversely designed metalens has high achromatic performance in the visible light region of four wavelengths and arbitrary polarization. Fu et al. [42] proposed a 2-bit encoding metasurface design method based on deep learning to perform an automatic matching of the metasurface structure and spectral response. Compared with traditional methods, this deep learning approach has higher efficiency and accuracy, which allows to increase the speed of the design cycle. Zhu et al. [43] designed a network that establishes the corresponding relationship between the focus features (i.e., amplitude and phase) and the geometric dimensions of the unit structure, which allows to accurately predict the unit structure of the metasurface.

In this paper, a high degree of freedom customizing the electric field method for metalens is proposed, where the adopted neural network is improved based on the pixGAN network. This improved pixGAN (I-pixGAN) designs the phase distribution of the metalens, and the constructed metalens (C-metalens) can manipulate the position of the focal point in three-dimensional space. Note that the target electric field image can be modified using existing electric field data, and it can even be drawn directly. The PB phase is then utilized to construct the metalens according to phase distribution.

Compared with traditional methods, our method of customizing the electric field is highly flexible and efficient and can significantly reduce the time required for metalens design. By precisely controlling the phase distribution of metalens, complex electric field control requirements can be achieved, and the difficulty of the design of other metalens complex functions can be simplified, such as the extended depth-of-focus metalens, holographic metasurfaces, and vortex beam metasurface. It can also promote the development of electric focusing systems and facilitate the automation and intelligence of optical systems.

## 2 Proposed method

**Figure 1:**
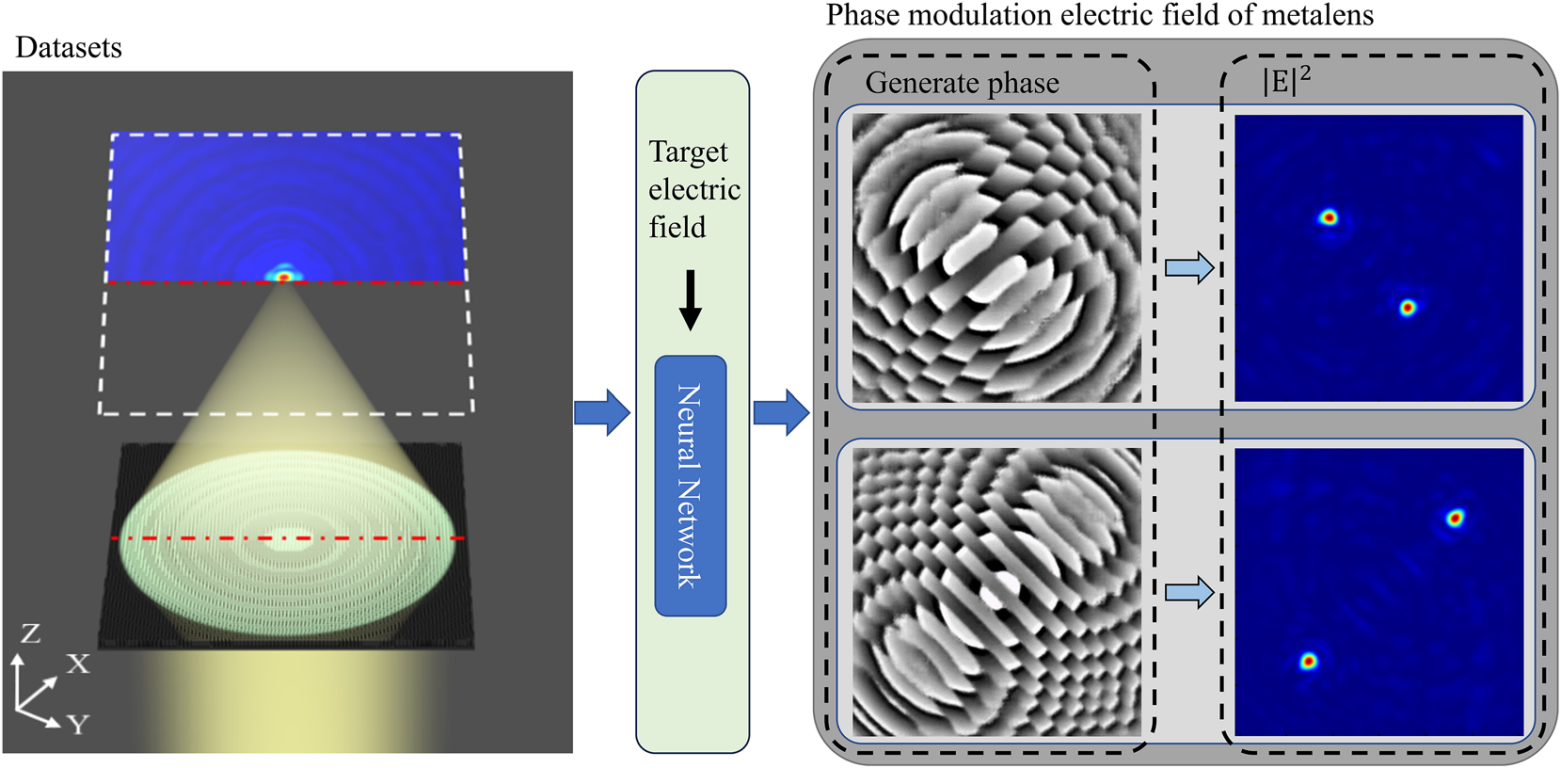
High degree of freedom control of electric field based on neural network. Using datasets for training, the neural network can generate phases based on the target electric field, which the metalens can modulate electric field to target one.

Based on the GAN network, an I-pixGAN was designed to generate the phase distribution of the metalens, and the electric field was focused at a specified position in three-dimensional space by the C-metalens, as shown in Figure 1. The network’s input is the electric field image at the focal plane, while its output is the image of the metalens phase. Due to the designed neural network, the proposed method does not rely on the phase requirements of the generalized Snell’s law in the continuous unit structure.

**Figure 2:**
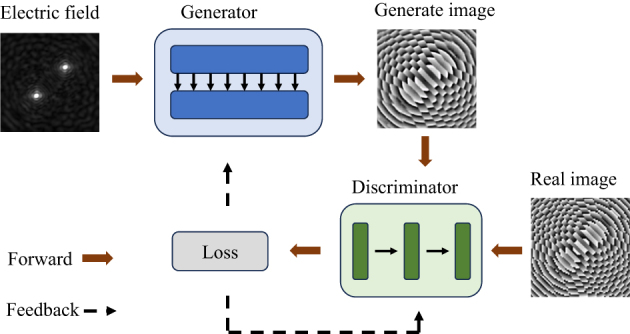
Schematic of the training and design process of the pixGAN network.

The GAN network is a generation network of high performance. It has significant potential for generating unknown images [44]. Since GAN network use adversarial training methods in game theory, the generated data are as close as possible to the real data in front of the discriminator. This paper expresses the electric field and phase distribution as images; therefore, an I-pixGAN based on the GAN network is adopted. The electric field and phase are two-dimensional matrices exhibiting a high correlation. Therefore, the I-pixGAN network is improved and a high-degree-of-freedom customizing electric field method for metalens is proposed. Our method uses the neural network to design a phase distribution of metalens based on the target electric field. The C-metalens can manipulate the focus of electromagnetic waves according to the customized target electric field. The target electric field can be customized by modifying the existing one or directly drawing it. The I-pixGAN network, consists of a generator and a discriminator, is developed based on the GAN network. In this study, it is implemented using the PyTorch architecture. The processed electric field image is input into the generator and discriminator for training. Finally, the network can generate a metalens phase image. The network development is shown in Figure 2. Note that the maximum focal length is 40 mm. In the grayscale image, when the pixel intensity is 40, the focus is not obvious. Therefore, the pixel value of the electric field in the figure is extended to the range of 0–255 to make it easier to observe the focus.

**Figure 3:**
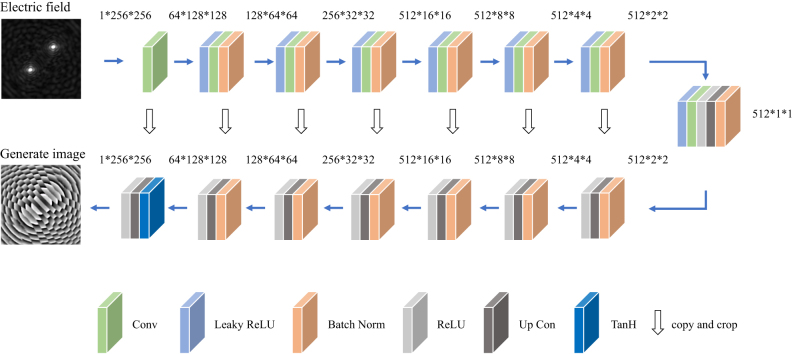
Generator network diagram.

In the original pixGAN [45], the input image and random noise should be input into the generation network. However, I-pixGAN achieved the unique electric field corresponding to the unique metalens phase distribution, and the proposed I-pixGAN does not require additional input random noise. This is due to the strict correspondence between the electric field and the phase distribution of the metalens, which results in additional random noise, interference, and network performance reduction. The generator used in this paper is based on the U-net architecture, which can retain more shallow information, such as the boundary information. It consists of 8 convolutional layers and 8 transposed convolutional layers, as shown in Figure 3. Note that the dropout is not used to provide noise. In addition, the generator only receives electric field images without introducing noise. This aims at extracting as many electric field features as possible while strengthening the correspondence between the input electric field and the output phase distribution.

**Figure 4:**
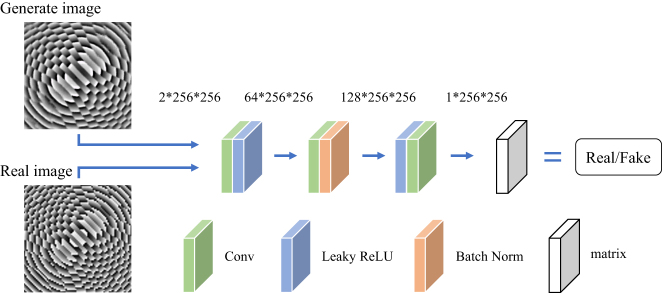
Discriminator network diagram.

The discriminator of the proposed pixGAN network, shown in Figure 4, consists of three convolutional layers. It outputs a matrix of size 1 × 256 × 256. The overall evaluation of the matrix is output as “true” or “false.” Since the shallow network can retain more detailed information, it has higher discrimination efficiency on the details of the metalens phase image [46].

The parameters of the generator and discriminator are taken from the study presented in [45]. They are provided in Supplementary information: Appendix A. A sigmoid + BCELoss loss function is adopted with a learning rate of 0.0002 and momentum parameters *β*_1_ = 0.5 and *β*_2_ = 0.999.

## 3 Results and analysis

### 3.1 Configuration of the datasets

In this study, we explore a universal methodology suitable for multiple frequency. In order to verify the reliability of our work, based on the unit structure and parameters given in [47], single-focus and double-focus metalens with frequencies of 110 GHz and 0.8 THz and focal lengths of 20–40 mm were constructed, respectively. The electric field datasets were procured through Lumerical, with the 110 GHz dataset comprising 5,921 data, and the 0.8 THz dataset containing 4,841 data. The electric field data consists of a 961 × 961 matrix.

**Figure 5:**
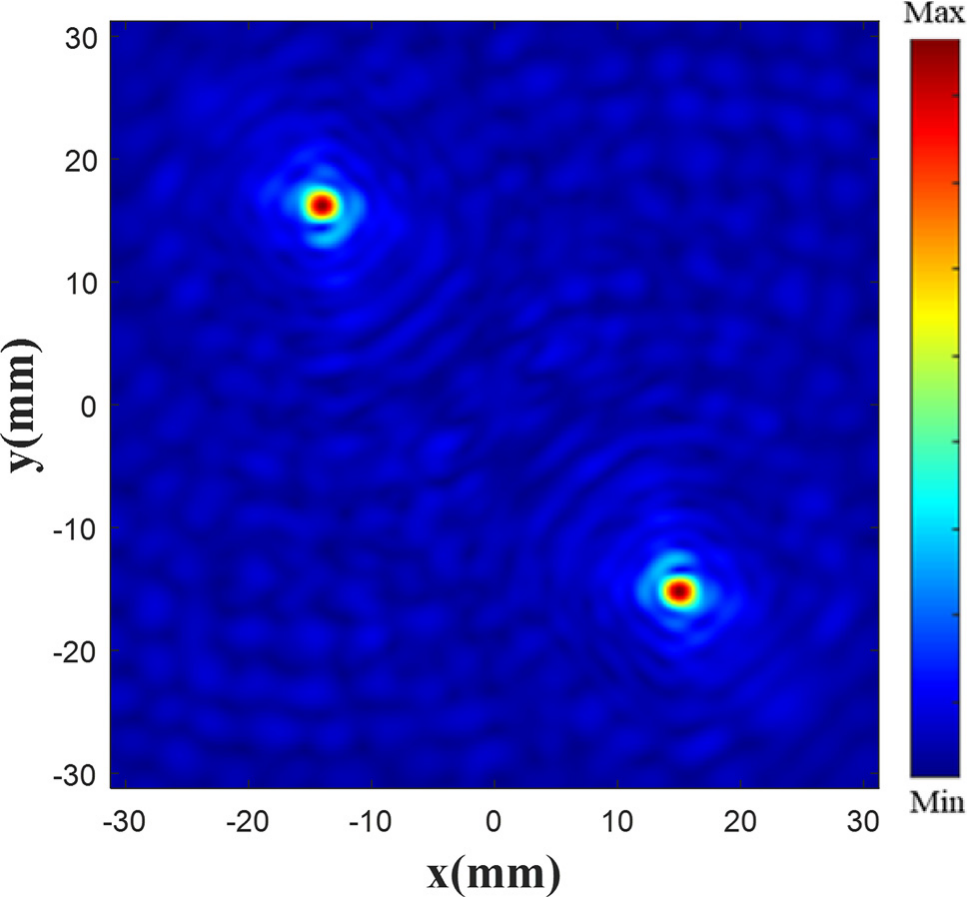
Electric field by Lumerical simulation.

The electric field data of the focus plane (i.e., the XY plane) are first extracted and considered as the training dataset, which includes focus and noise data. This dataset comprises multiple focal lengths, and the underlying performance varies with the focal length and focus type. Therefore, the electric field data are normalized to retain the distribution characteristics. Afterward, the normalized data are multiplied by the focal length to distinguish the electric field data at different focal lengths. Finally, the data are converted into a grayscale image, which is considered as the input of the network. Because the normalized data consist of a matrix comprising values in the range of 0–1, the effective focal lengths are between 0 mm and 255 mm. Figure 5 shows the electric field obtained by Lumerical simulation with a focus of 20 mm.

The computation of the calculate phase in the datasets is based on the study presented in [47]. The phase distribution of the metalens is divided into two modes: single focus and double focus.

The phase of the single focus metalens is calculated as:

(1) $$\begin{array}{c} \varphi \left(r\right)=\frac{2\pi }{\lambda }\left(\sqrt{r^{2}+f^{2}}-f\right) \end{array}$$

where $r=\sqrt{x^{2}+y^{2}}$ , $\left(x,y\right)$ is the unit structure position, and *f* is the focal length.

The phase of the double focus metalens is calculated through the superposition of multiple single-focus metalens phases [5], [42]:

(2) $$\begin{array}{c} \varphi_{A}=\frac{2\pi }{\lambda }\left(\sqrt{x_{A}^{2}+y_{A}^{2}+f^{2}}-f\right) \end{array}$$

(3) $$\begin{array}{c} \varphi_{B}=\frac{2\pi }{\lambda }\left(\sqrt{x_{B}^{2}+y_{B}^{2}+f^{2}}-f\right) \end{array}$$

(4) $$\begin{array}{c} \varphi_{\text{total}}=\arg\left[\exp\left(i\varphi_{A}\right)+\exp\left(i\varphi_{B}\right)\right] \end{array}$$

where *φ*_*A*_ and *φ*_*B*_ are the phase distributions of metalenses A and B, respectively.

**Figure 6:**
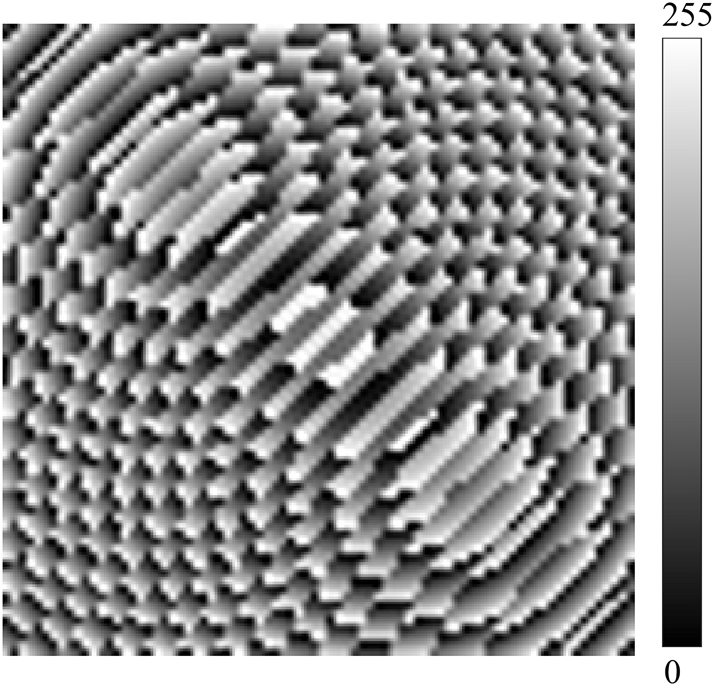
The phase image after processing.

In the dataset, the number of the metalens unit structures is 80 × 80, and the phase distribution is a matrix of size 80 × 80, which is converted into an image, where the phase of each unit structure corresponds to a pixel. The computed phases are in the range of −180 to 180. Since the pixel intensities in the grayscale image should be in the range of 0–255 (i.e., each pixel is represented by 8 bits), the phase data range is first translated to 0–360 and then scaled to 0–255. The data processing method used to determine the value after scaling (*D*_*t*_) are expressed in Equation (5). Finally, the data are converted into grayscale images. The phase image after processing is shown in Figure 6.

(5) $$\begin{array}{c} D_{t}=\frac{D*S_{t}}{S_{p}} \end{array}$$

where *D* is the original value, *S*_*p*_ and *S*_*t*_ are the maximum data before and after scaling, respectively.

Due to the fact that the phase image and the electric field image have different parameters, the former has a size of 80 × 80 and its values are in the range of 0–360, while the latter has a size of 961 × 961, and its values are much less than 255. Therefore, the electric field image is scaled down to the size of 256 × 256 image, and the phase image is expanded to the size of 256 × 256 using bicubic interpolation. The phase image of small size and high values is expanded and the electric field image of large size and low values is scaled to retain the data features as much as possible, and thus facilitate the network training.

### 3.2 Experimental verification

**Figure 7:**
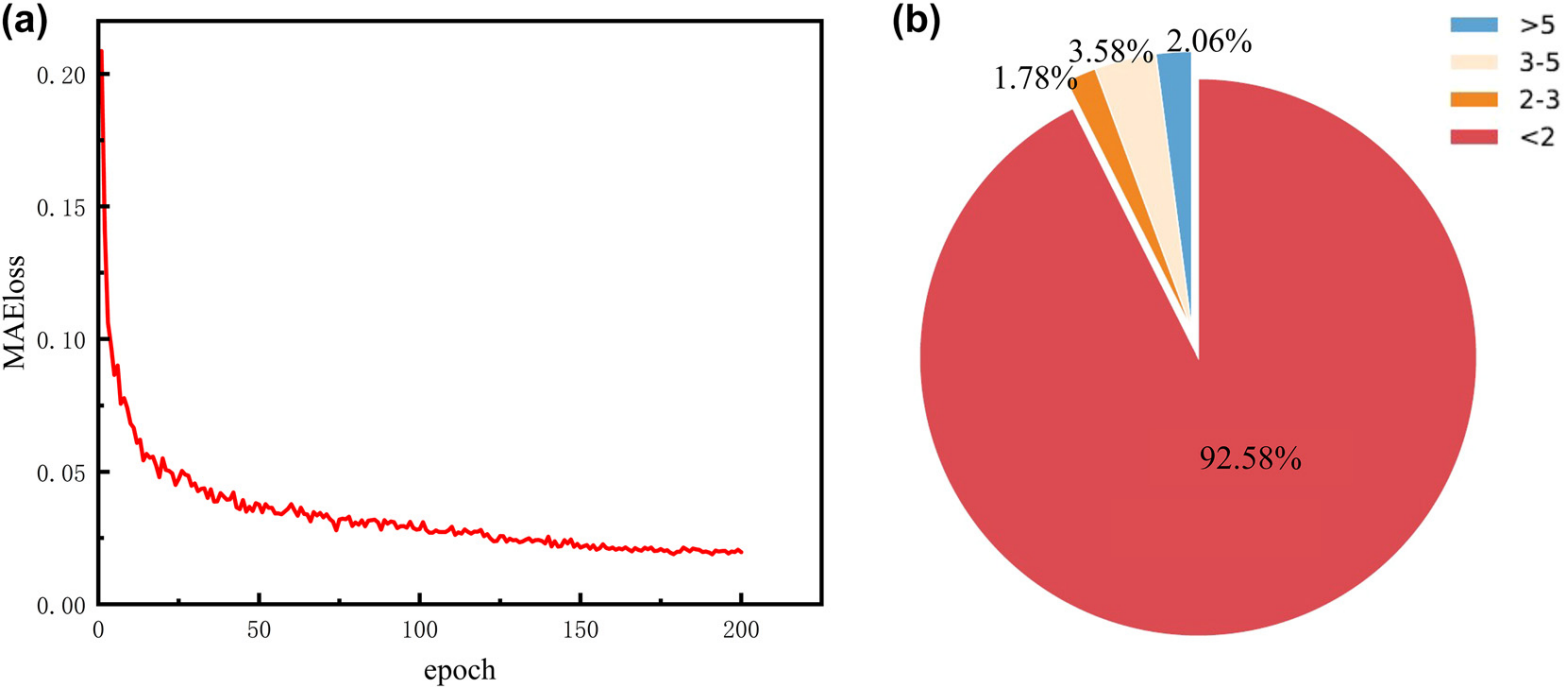
Training error diagram. (a) Shows the change of MAEloss with training, and (b) shows the verification of AEEP results. Red means AEEP is less than 2, orange means AEEP is between 2 and 3, light pink means AEEP is between 3 and 5, and blue means AEEP is greater than 5.

In order to verify the stability and convergence of the network, as shown in Figure 7(a), we processed the Mean Absolute Error loss (MAEloss) of the generated image and the original image. After 150 epochs, the network training tended to be stable, and the MAEloss value was about 0.02. We used the 200th epoch for independent verification experiments, and the results are shown in Figure 7(b), which uses 50 sets of datasets for Absolute Error of Each Pixel (AEEP) verification. The results show that the area where the AEEP between the generated image and the existing image is less than 2 accounts for about 92.58 %, so the metalens constructed using the generated phase can modulate the target electric field. The detailed values of the cross-sectional electric fields generated by our method and the traditional method can be found in the Supplementary information: Appendix B.

A customized target electric field region was used to verify the proposed network’s capability and evaluate its ability to customize the electric field. First, verify that the target electric field is customized by modifying the existing electric field. The focal plane electric field data at 20 mm are normalized and then multiplied by 40, since the focal length of the target electric field is equal to 40 mm. Afterward, the obtained data are scaled to a matrix of size 256 × 256. This matrix is converted to a grayscale image, where each pixel denotes the corresponding matrix value. The grayscale image is input into the network, and another one, having a phase image size of 256 × 256 and pixel values in the range of 0–255, is generated. The pixel values are then scaled to the range of 0–360 and converted to a matrix comprising values between −180 and 180. Finally, the 256 × 256 matrix is isometrically reduced to another one of size 80 × 80, which denotes the scale of the metalens unit structure. This matrix is considered as the phase distribution of the target metalens.

**Figure 8:**
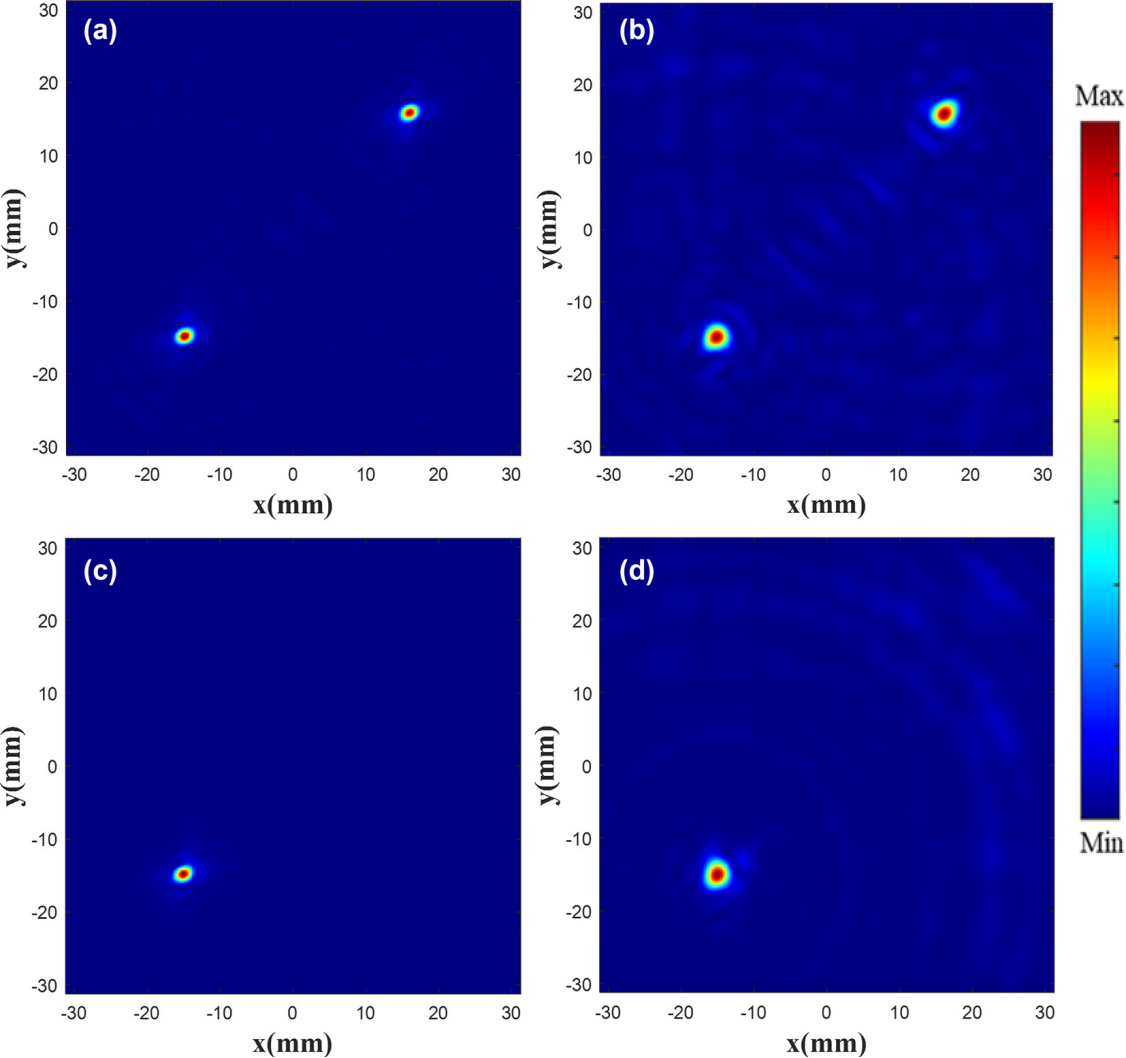
|*E*|^2^ diagram of the metalens. Existing double focus (a) and single focus (c) |*E*|^2^ diagram. Customized double focus (b) and single focus (d) |*E*|^2^ diagram.

According to the PB phase, the rotation angle of the unit structure is half that of the phase. The metalens is arranged according to the phase matrix. Its parameters are similar to those presented in [47] and its working frequency is 110 GHz. The |*E*|^2^ diagram of the metalens is shown in Figure 8, where Figure 8(a) and (c) is the existing electric field with the focus of 20 mm, and (b) and (d) is customized |*E*|^2^ (metalens constructed using our method). As the existing data contain part of the target electric field characteristics, a high performance is obtained, and the focus position and shape are highly consistent with the original electric field. In Figure 8, at the focus plane, the double focus coordinates are (−15 mm, −15 mm), (16 mm, 16 mm), and the single focus coordinates are (−15 mm, −15 mm). Theoretical simulation results can be found in Appendix C. The results indicate that the focal point of the electric field can be controlled to the custom position, and the FWHM (full width at half maximum) of the custom electric field is 18.18 % greater than the theoretical electric field.

**Figure 9:**
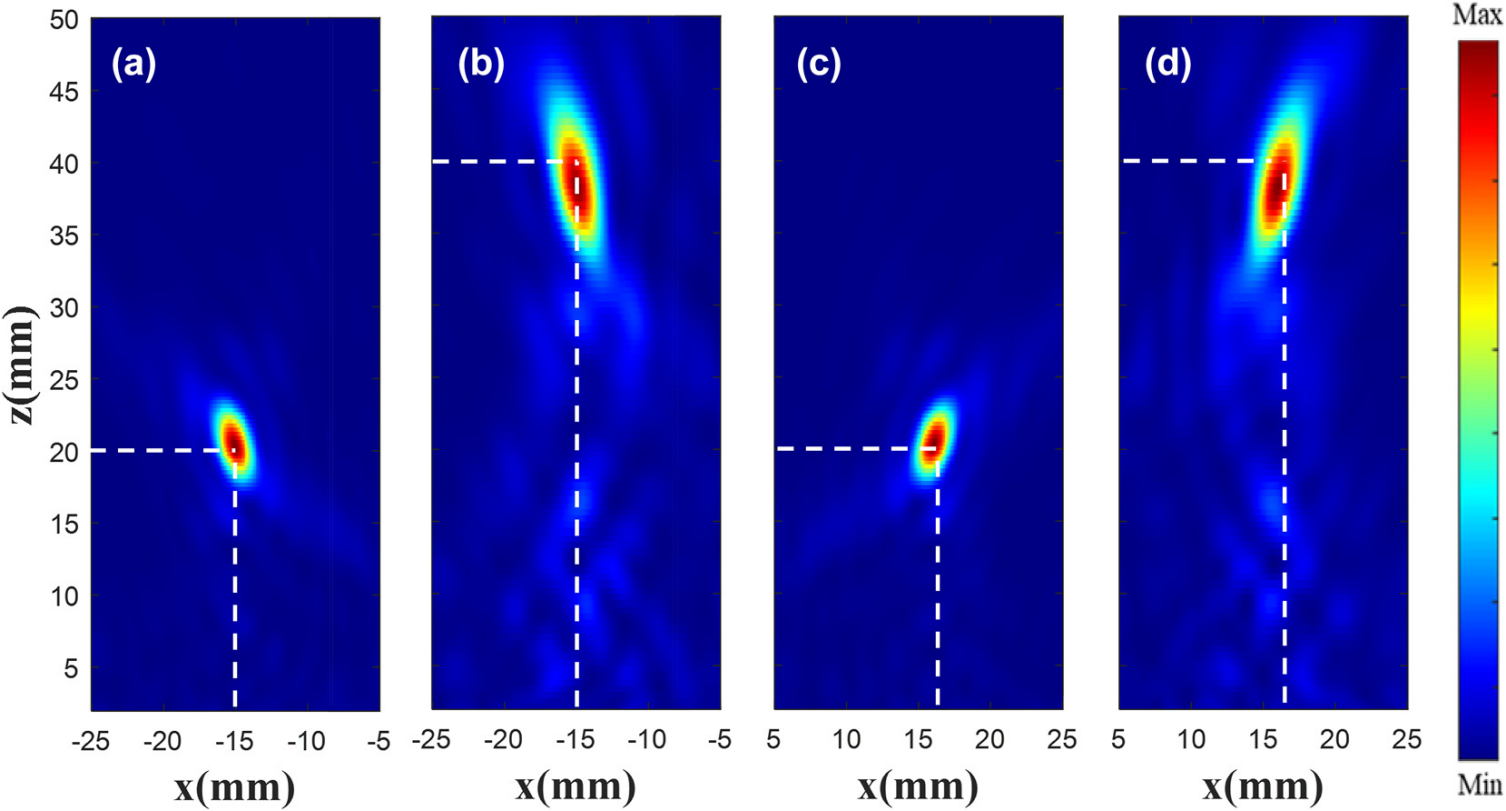
|*E*|^2^ diagram at each focus. Existing (a) and customized (b) |*E*|^2^ diagram at the focus (−15 mm, −15 mm). Existing (c) and customized (d) |*E*|^2^ diagram at the focus (16 mm, 16 mm).

In order to observe the customized double focus |*E*|^2^, monitors are placed at *x* = 0, *y* = −15 mm, and *x* = 0, *y* = 16 mm, shown in Figure 9. Figure 9(a) and (c) is the existing |*E*|^2^ diagram, and (b) and (d) is the customized |*E*|^2^ diagram. The results show that the two focus points are focused around *z* = 40 mm, the customized electric field has the highest focus intensity at *z* = 38.5 mm, and the focus position offset is less than one wavelength.

**Figure 10:**
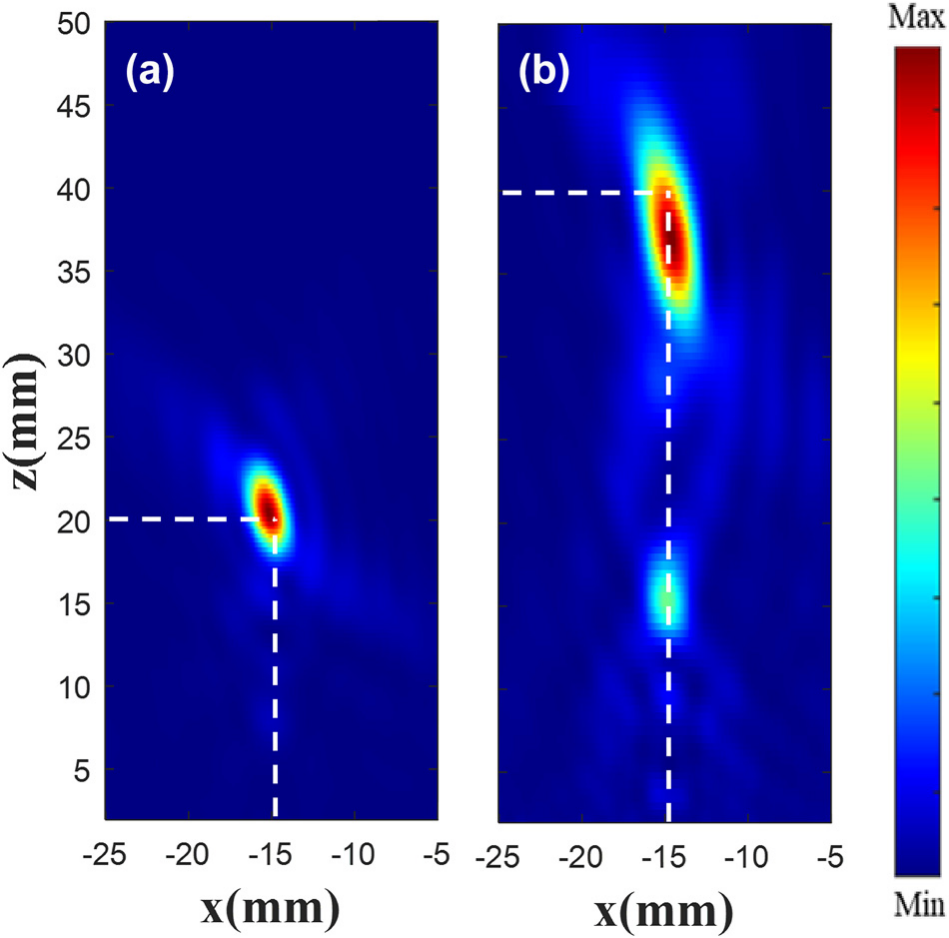
|*E*|^2^ diagram at focus. Existing (a) and customized (b) |*E*|^2^ diagram at the focus (−15 mm, −15 mm).

In order to observe the customized single focus |*E*|^2^, a monitor is placed at *x* = 0, *y* = −15 mm, and the results are shown in Figure 10. Figure 10(a) is the existing |*E*|^2^ diagram, and Figure 10(b) is the customized |*E*|^2^ diagram. The results show that a focus is focused around *z* = 40 mm, the metalens constructed using the generated phase has the highest focus intensity at *z* = 38 mm, and the focus position offset is less than one wavelength.

Next, customize the electric field by drawing directly. An image of size 256 × 256, having pixel values of 0, is first constructed. Then draw the circle in the image that corresponds to the size of the focus in the existing electric field. This circle is considered as the focus, and its gray level value presents the focal length in the electric field. Finally, the image is saved as grayscale and input into the network as draw target electric field.

**Figure 11:**
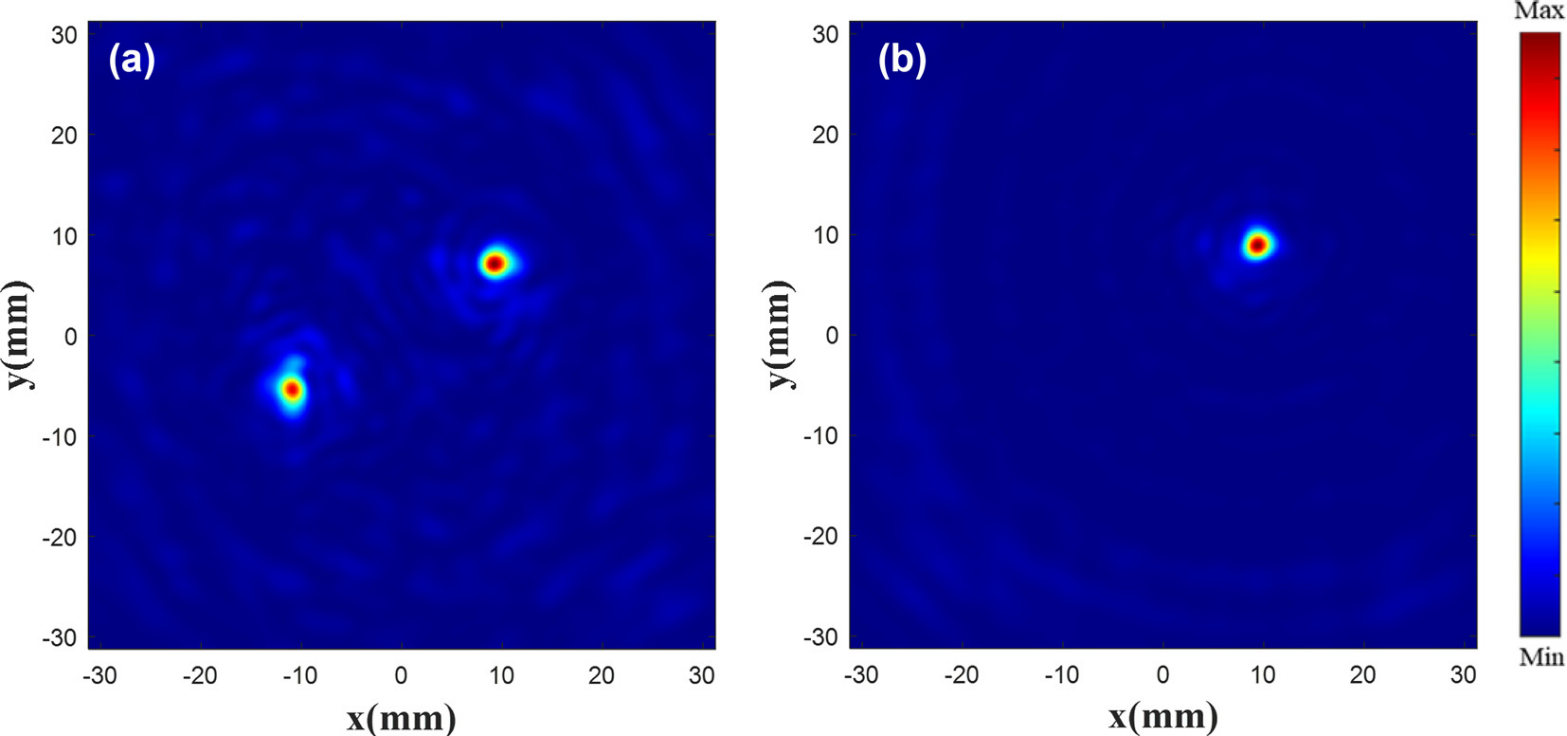
|*E*|^2^ diagram of the metalens. |*E*|^2^ diagram of double focus (a) and single focus (b) at the focal plane.

During verification, an image of 256 × 256 pixels is considered and its background is set to 0. Single or double circles are then drawn at random positions on the canvas with a gray value of 30 (i.e., the focal length of the target electric field is set to 30 mm). This grayscale image is saved and input into the network, and the generated phase image is processed in the same way as the phase image of the modifying the existing electric field. The unit structure of the metalens is arranged according to the generated phase. The |*E*|^2^ diagram of the metalens is shown in Figure 11. It can be seen that a difference exists between the distributions of the drawn target electric field and the original electric field. This is due to the fact that the former is created by directly clicking with the brush tool. For example, the focus edge and focus shape of the target electric field vary with the brush movement.

**Figure 12:**
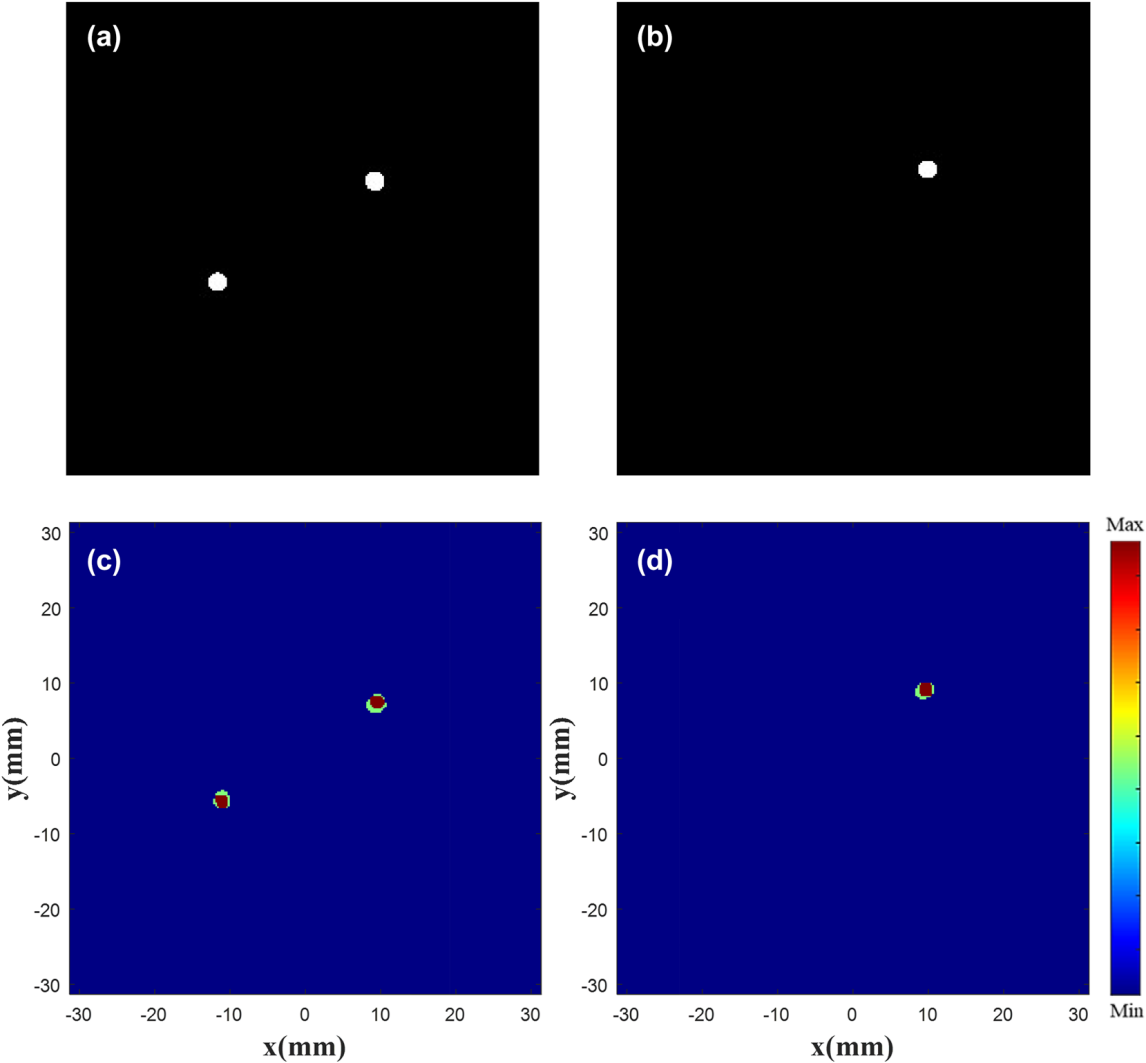
Focus contrast diagram. Drawn double focus (a) and single focus (b) |*E*|^2^ diagram. Superimposed double focus (c) and single focus (d) normalized *|E|* ^*2*^ diagram.

Figure 12 shows difference of the focus position between the drawn one and the generated one. Figure 12(a) and (b) is a random drawn electric field diagram, where the image is processed in the same manner as the electric field image in Figure 4. Figure 12(c) and (d) is the superposition of the customized normalized |*E*|^2^ and the drawn one. The superposition of normalized |*E*|^2^ shows that regardless of single or double focus, the customized results are nearly within the bounds of the drawn one, and the focus position offset is less than half a wavelength. More simulation results in Supplementary information: Appendix C.

**Figure 13:**
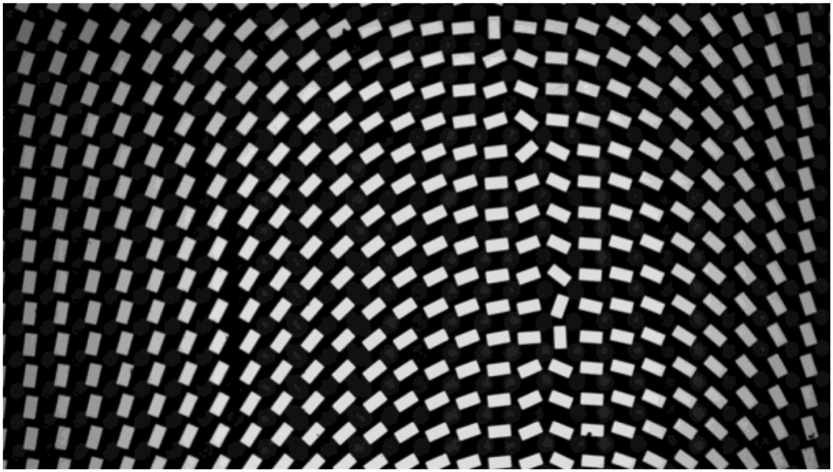
Optical microscope image of the partial metalens.

To test the feasibility of our method in other frequency, we repeated the 110 GHz experiment at the 0.8 THz. Figure 13 shows the optical image of the prepared metalens. The unit structure is a rectangular column with *W* = 42 µm, *L* = 80 µm, *H* = 40 µm, and the period of the unit structure is 110 µm.

**Figure 14:**
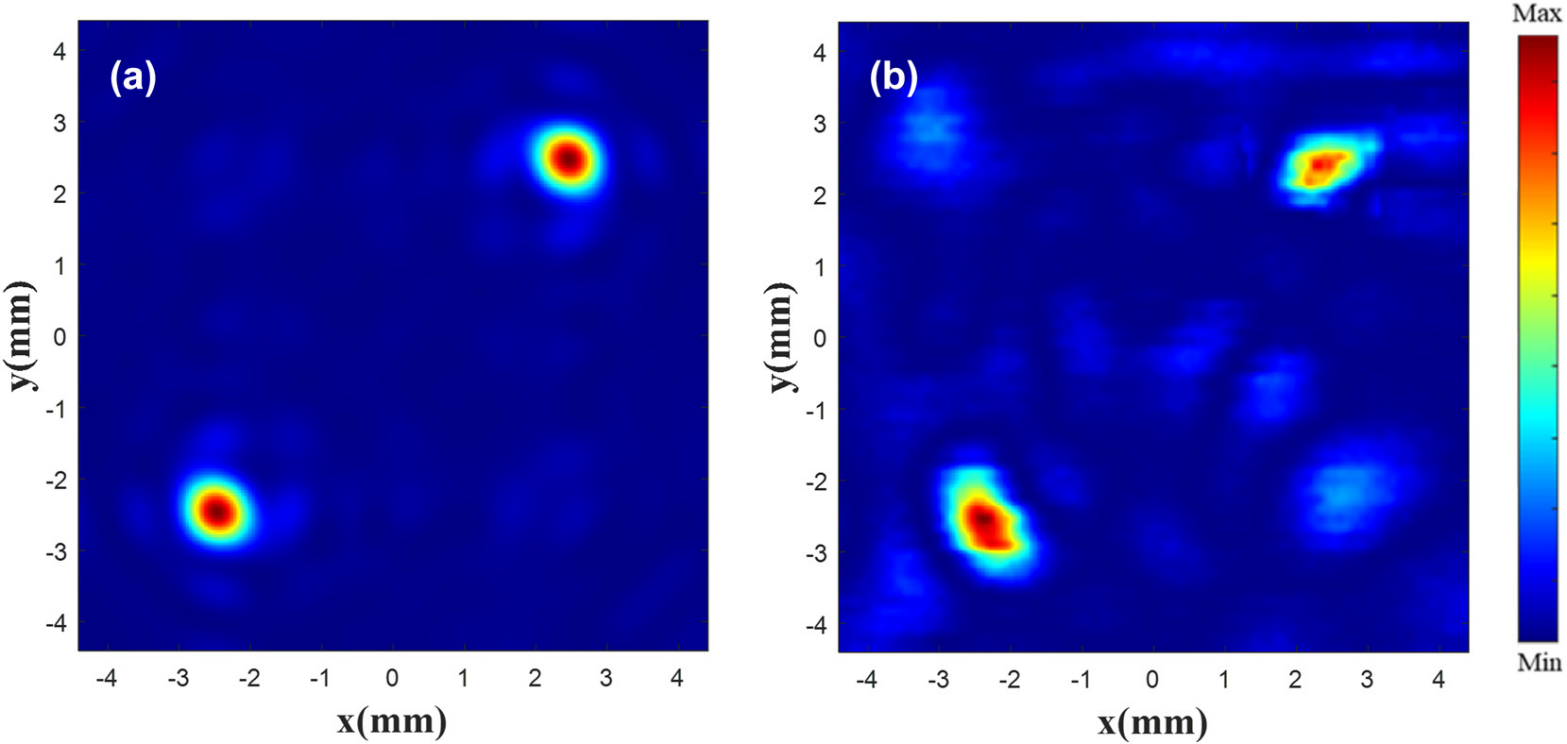
Focus contrast diagram. Existing (a) and customized (b) *|E|* ^*2*^ diagram at the focus.

The electric field of the metalens was imaged using a terahertz time-domain spectroscopy system. We customized an electric field with a focus of 20 mm using existing electric field with the focus of 15 mm. The neural network designed a metalens based on the customized electric field. Figure 14 below shows the results of the metalens. At *z* = 20 mm, the metalens designed by the neural network can focus on the specified position. Compared with the existing electric field, the intensity of the two foci of the customized electric field is inconsistent. This can be attributed to the inconsistent uniformity of the processing.

**Figure 15:**
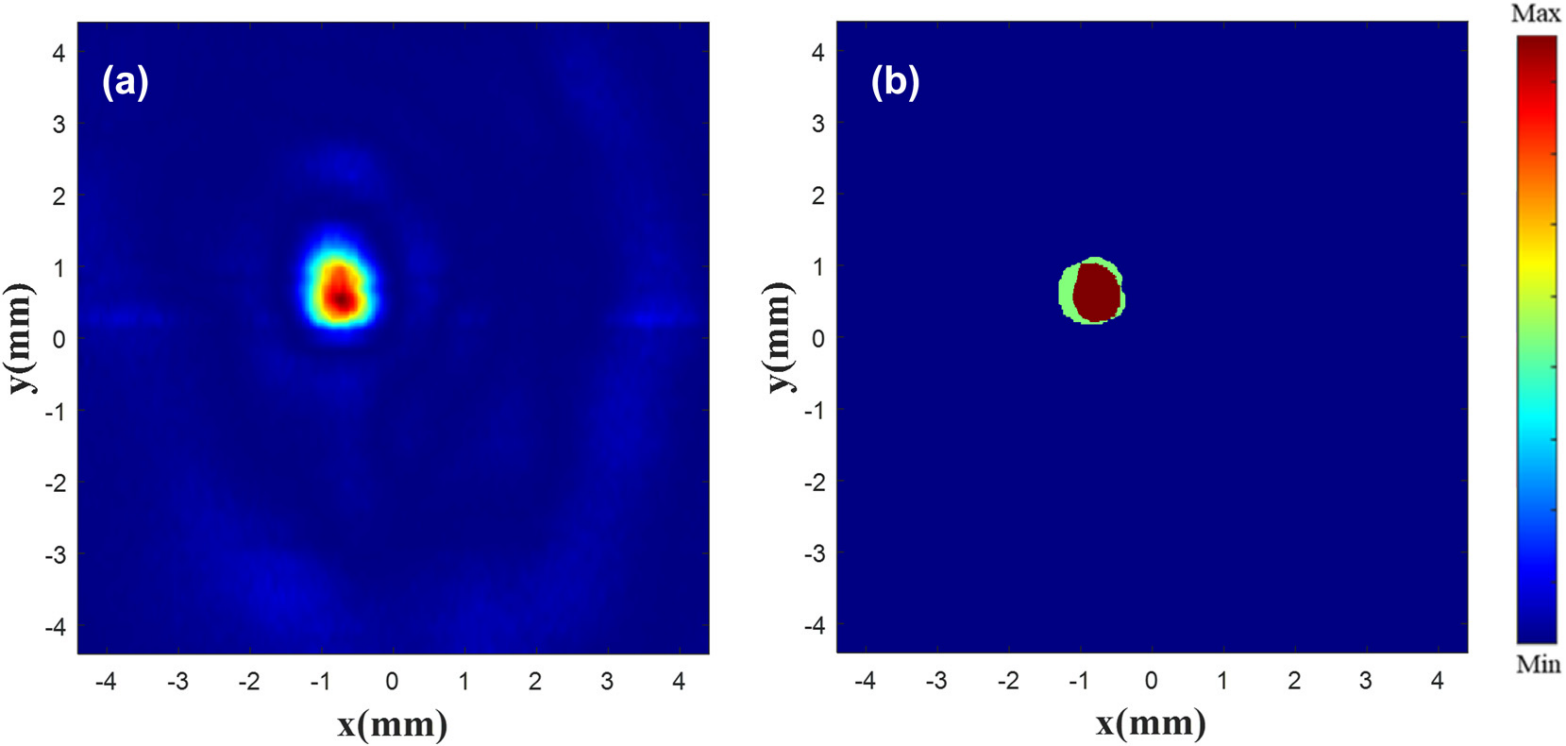
Focus contrast diagram. Drawn single focus (a) *|E|* ^*2*^ diagram. Superimposed single focus (b) normalized *|E|* ^*2*^ diagram.

We draw a focus arbitrarily on a surface with a focal length of 15 mm. Figure 15 below is a comparison of the measured electric field and the drawn position. The experimental results show that the drawn point is at the same position as the actual focus. Moreover, compared with the simulation results at 110 GHz, the drawn point and the focus can be highly coincident. We believe that the drawn focus can be correctly focused to any position, and the focus offset is less than one wavelength. More experimental results in Supplementary information: Appendix E.

The proposed method for the customization of the electric field with high degree of freedom of the metalens is feasible. The existing metalens use the continuous phase matching equation to calculate the phase, which lacks flexibility. The proposed method is able to solve this problem. The phase of the unit structure can perform modulation matching within a large range, which allows the joint modulation of multiple unit structures and the compensation for each other, rather than being independently modulated by a certain unit structure. Moreover, this flexible control method can reduce the modeling requirements at the simulation level and greatly shorten the design time of the metalens. In some cases, the focus position can be accurately determined by eye without the need of a coordinate system. In this case, the metalens phase can be directly obtained by drawing, which significantly reduces the design difficulty and increases the degree of freedom in the modulation of the focus. This also provides solutions for other subsequent applications of metasurface. In this paper, an I-pixGAN can design the phase distribution of the metalens based on the customized target electric field. Compared with existing end-to-end inverse designs, we have achieved focus control in three-dimensional space and simplified the method of constructing input data. This is a high-degree-of-freedom electric field manipulation method. When constructing the target electric field, existing electric fields or drawn one can be used to control the single/double-focus positions. In future work, combined with tunable metasurfaces, electric focusing can be achieved, facilitating the automation of optical and terahertz systems.

## 4 Conclusions

This paper proposes a method for the customization of electric fields. Based on the customized target electric field, our I-pixGAN designs a phase distribution of the metalens, and the electric field is focused by the C-metalens at a specified position in three-dimensional space. The electric field and metalens phase are converted into grayscale image processing, and the data are replaced by pixels of the image. The existing electric field is then normalized and multiplied by the target focal length, or an “electric field” image is manually drawn. The proposed network can design a phase distribution of the metalens, and the electric field can be modulated into the shape of the target electric field by the C-metalens. Experimental results show that our method can effectively customize the electric field with high degrees of freedom by using neural networks to design the phase of the metalens. The focus position offset is within one wavelength with the FWHM higher than 18.18 % of the theoretical value. Moreover, the focus can be customized at the target position using our method. It is worth noting that both the 100 GHz and 0.8 THz datasets can achieve customization of the electric field with high degrees of freedom, and the frequency does not affect the performance of I-pixGAN. Finally, neural networks can not only be used to modulate more complex electric field but also promote the automation and intelligence of optical/terahertz systems, such as electric focusing.

## Supplementary Material

Supplementary Material Details
