# Supplementary material for: Customizing the electric field of metalens with high degrees of freedom based on neural network
Source: Nanophotonics. 2025 Apr 14;14(12):2187–98. doi: 10.1515/nanoph-2025-0032 (PMC12147552; doi:10.1515/nanoph-2025-0032)
Supplement: Supplementary file 1 — Supplementary Material Details [file j_nanoph-2025-0032_suppl_001.docx]

Supplementary Information:

Customizing the electric field of metalens with high degrees of freedom based on neural network

Quansheng Zhang, Di Guo, Changsheng Shen, Zhaofu Chen, Hehong Fan, Changgui Lv, Qilong Wang and NingFeng Bai *

School of Electronic Science and Engineering, Southeast University, Nanjing 210096, China

bnfeng@seu.edu.cn

**Abstract:** The metasurfaces are able to flexibly control electromagnetic waves. However, their design necessitates strict phase matching, which requires high computational load, and its control method has low flexibility. This paper proposes a novel method for the customization of the electric field of metalens with high degrees of freedom based on an improved Pixel Generative Adversarial Network (I-pixGAN). This I-pixGAN can design the phase distribution of the metalens according to the customized target electric field, and the constructed metalens based on this phase can modulate the target electric field. The results show that the proposed I-pixGAN can arbitrarily control the position of the focus in three-dimensional space, where the focus position is offset within one wavelength. Therefore, we do not need to strictly follow the electric field distribution characteristics, and can flexibly construct unknown electric field, realizing a highly free electric field customization method in three-dimensional space, providing a new way for electric field operation. This paper promotes the development of electric focusing systems, not only improving focusing accuracy, but also enhancing system flexibility and promoting the automation and intelligence of optical and terahertz systems.

**Appendix A. Network details and training equipment**

The considered neural network consists of a generator and a discriminator. The generator contains eight convolutional layers (1, 64, 128, 256, 512, 512, 512, and 512 input channels or feature maps) and eight transposed convolutional layers (512, 1024, 1024, 1024, 1024, 512, 256, and 128 input channels or feature maps), while the discriminator contains three convolutional layers (2, 64, and 128 input channels or feature maps). The input channel becomes larger, due to the fact that the transposed convolutional layer incorporates the upsampled data from the previous level and merged data from the corresponding convolutional layer.

The Leaky Relu layer is used before the next convolution layer and not directly after the first convolution of the generator. Batch normalization is used after transposing the convolution layer, and Relu activation is adopted before the next transposed convolution. The output data of the first convolution layer are fused with those obtained by the last layer of transposed convolution. The second convolution and the penultimate transposed convolution feature are also fused. The data processing sequences of the fusion are basically the same. Except for the first convolution, the sequence of the other layers is activation-convolution-normalization. In addition, Relu activation is used before the last transposed convolution, and TanH activation is used after the transposed convolution. The generator is shown in Figure 5. After three convolutions, the discriminator generates a matrix of size 1 × 256 × 256. During this period, Relu activation is used after the first convolution, Batch normalization is performed after the second convolution, and Relu activation is finally used before convolution. The discriminator is shown in Figure 6.

The training of the model was performed on a win10 operating system with NVIDIA RTX 4090.

**AppendixB. Performance comparison**

Figure S1 (a) shows the metalens customized by our method, and Figure S1 (b) shows the metalens by the traditional method. Comparing the maximum value of the focus, our method is about 15.80% lower than the traditional method.


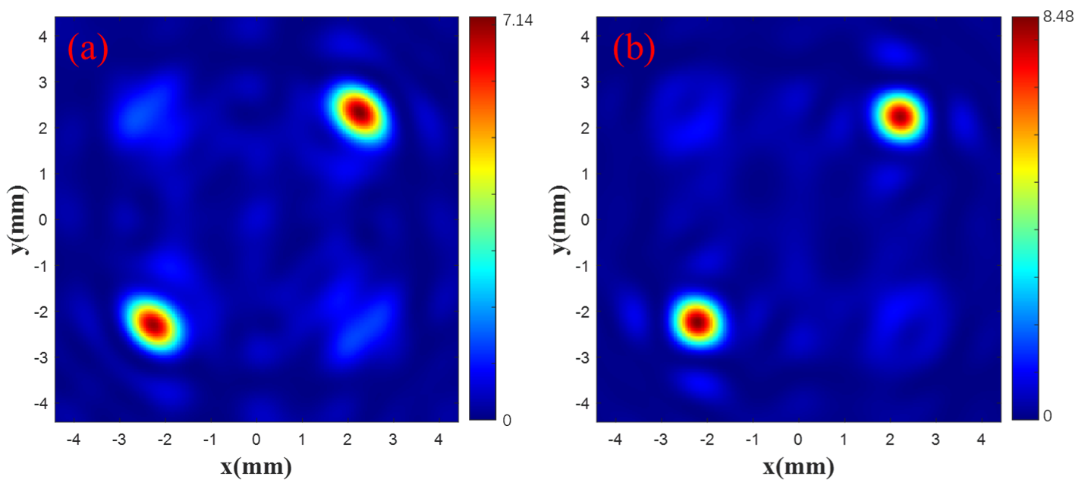


Figure S1. |*E*|^2^ diagram of the metalens. Customization focus (a) and Traditional focus (b)

**Appendix C. Supplementary simulation results**

Figure S2 shows the use of theoretical design for the metalens, with the design focus at z=40mm. The position of the focal point is the same as the metalens at the focal point of 20mm.


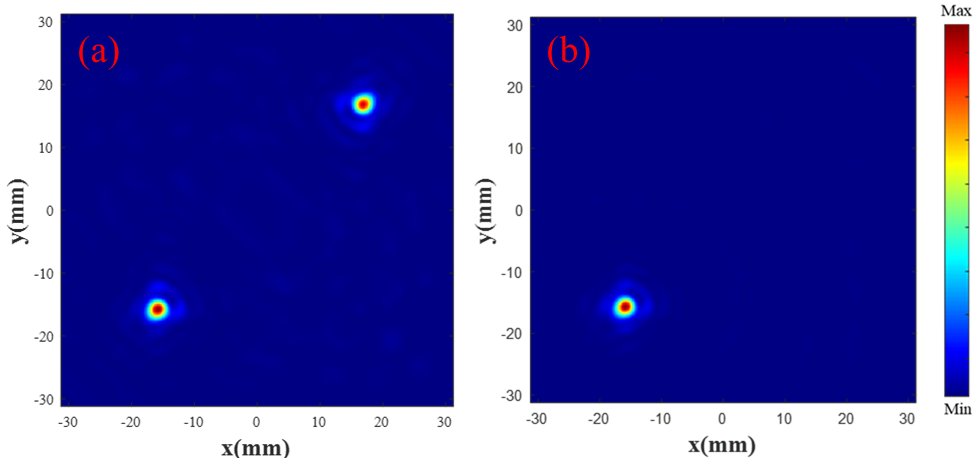


Figure S2. |*E*|^2^ diagram of the metalens. Theoretical double focus (a) and single focus (b).

Figure S3 (a) and (b) show the customization of the target *|E|^2^* by modifying electric field at a focal length of 30 mm. Figure S3 (c) and (d) show the customization of the target *|E|^2^* by drawing electric field at a focal length of 40 mm.


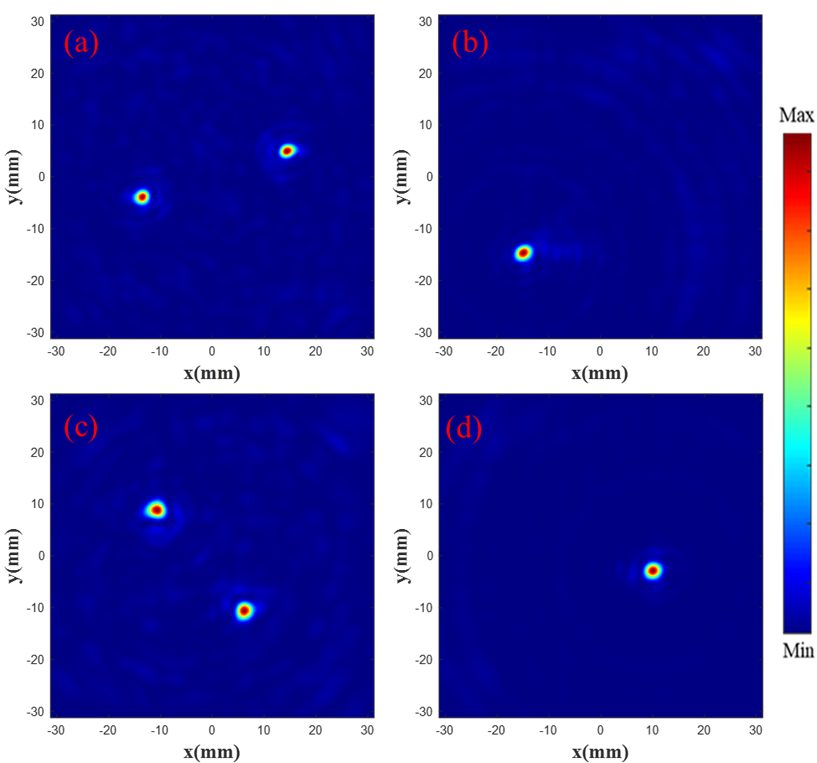


Figure S3. *|E|^2^* diagram of the metalens. (a) and (b) are *|E|^2^* diagrams customized by modifying electric field. (c) and (d) are *|E|^2^* diagrams customized by drawing electric field.

Figure S4 is a cross-sectional view of the focal point, showing the cross-sections of each focal point in Figure S3.


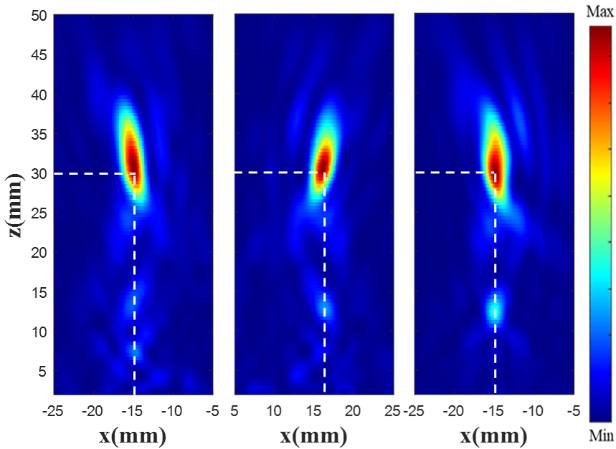


Figure S4. *|E|^2^* diagram at each focus. Customized double focus *|E|^2^* diagram at the focus (-15 mm, -15 mm) (a) and (16 mm,16 mm) (b). Customized single focus (c) *|E|^2^* diagram at the focus (-15 mm, -15 mm).

Figure S5 shows the simulation results of the drawing electric field at z=40mm.


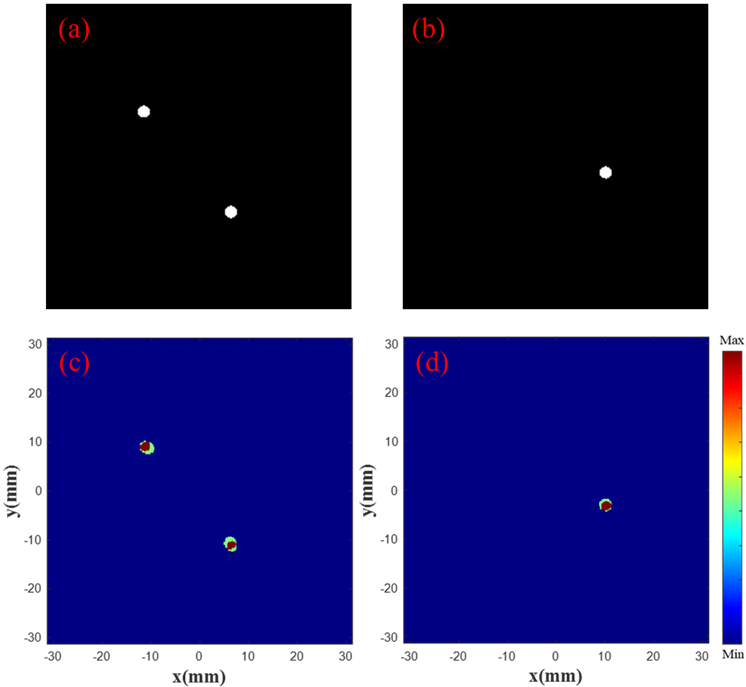


Figure S5. Focus contrast diagram. Drawn double focus (a) and single focus (b) electric field diagram. Superimposed double focus (c) and single focus (d) normalized *|E|^2^* diagram.

**Appendix D.** **Metalens fabrication**

**
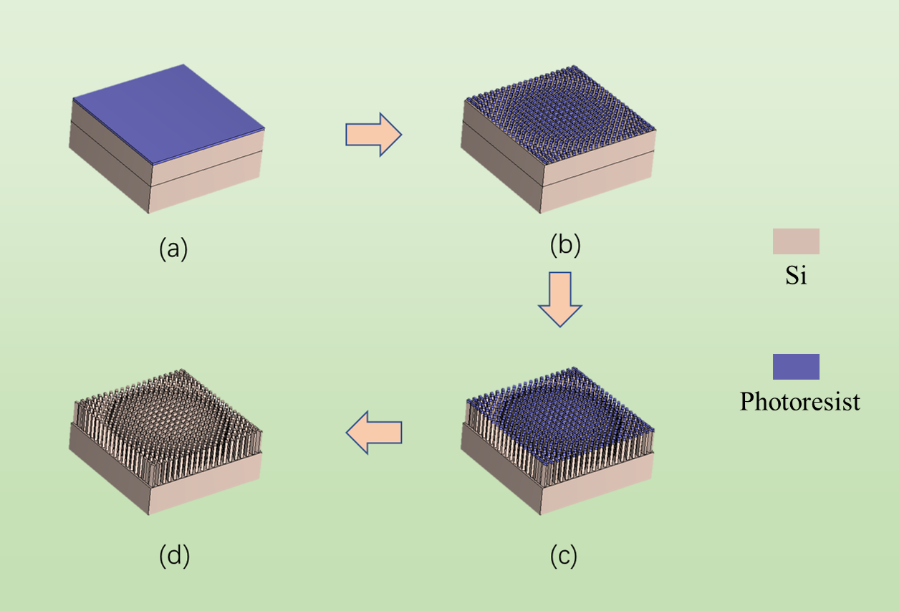
**

Fig. S6. Schematic showing the major steps of the fabrication process.

As shown in Figure S6, there are four steps in the manufacturing process of metalens: (a) photoresist coating. (b) Photolithography produces patterns of photoresist. (c) Reactive ion etching method for etching. (d) Except for photoresist.

**Appendix E. Supplementary experiment results**

Figure S7 is a customized electric field diagram modified from the existing 20 mm focal electric field to 15 mm.


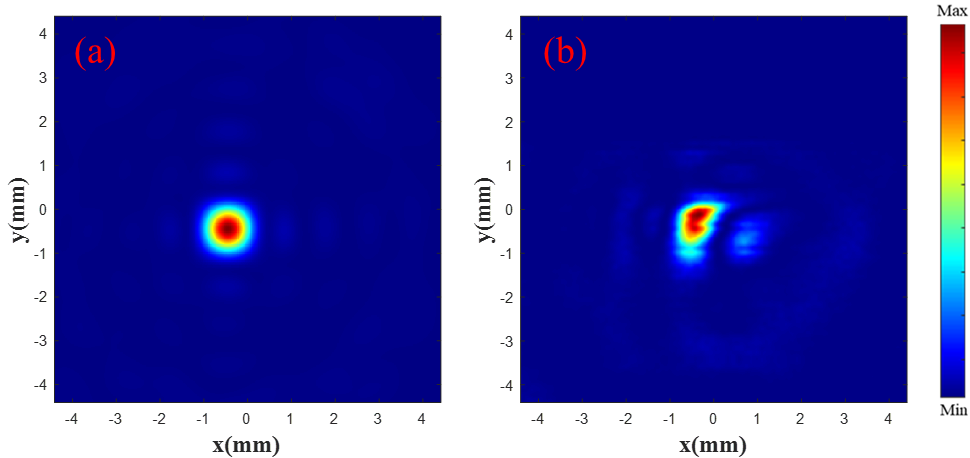


Figure S7. *|E|^2^*diagram of the metalens. Existing (a) and customized (b) *|E|^2^* diagram at the focus.

Figure S8 shows the result of arbitrarily drawing the electric field at z=20mm.


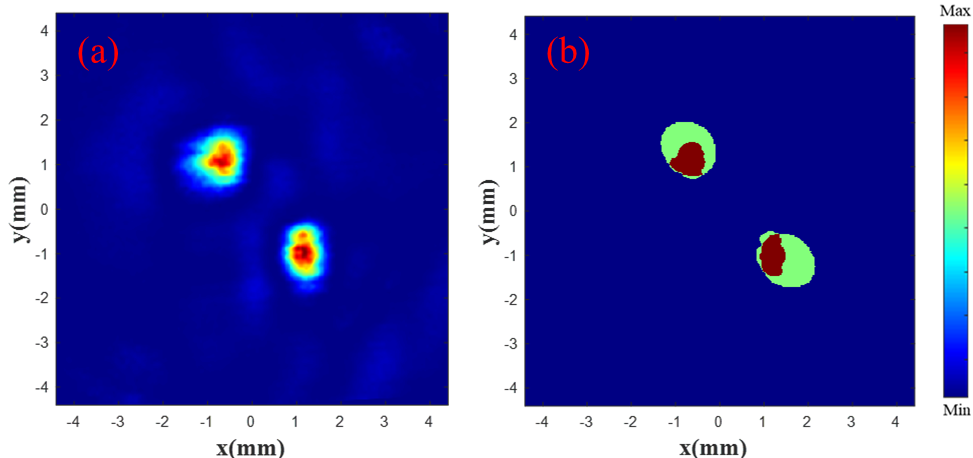


Figure S8. Focus contrast diagram. Drawn single focus (a) *|E|^2^* diagram. Superimposed single focus (b) normalized *|E|^2^* diagram.
